# Energy and Real Space Characteristics of Non‐Covalent Interactions Across the Periodic Table

**DOI:** 10.1002/jcc.70268

**Published:** 2025-11-24

**Authors:** Eline Desmedt, Katarzyna Zator, Tatiana Woller, Roberto A. Boto, Mercedes Alonso, Julia Contreras‐García

**Affiliations:** ^1^ Vrije Universiteit Brussel, ALGC Brussels Belgium; ^2^ Laboratoire de Chimie Théorique Sorbonne Université and CNRS Paris France; ^3^ Donostia International Physics Center University of the Basque Country Donostia‐San Sebastián Spain; ^4^ CNRS Paris France

**Keywords:** EDA, halogen bonding, NCI, non‐covalent interactions, Pnictogen bonding

## Abstract

Weak hydrogen bonds, pnictogen bonds, and halogen bonds are examined through comprehensive energy decomposition and electron density topology analysis to establish unified characterisation criteria for non‐covalent interactions. Energy decomposition analysis reveals that these interactions exist along a continuum defined by the relative contributions of electrostatic, orbital, and dispersive components, with linear hydrogen bonds exhibiting predominantly electrostatic character, π‐hydrogen bonds showing balanced orbital‐dispersion contributions, and pnictogen bonds demonstrating dispersion‐dominated behaviour similar to lighter halogen systems. Non‐covalent interaction (NCI) analysis provides a unifying framework where interaction character correlates systematically with spatial distribution: dispersive interactions generate extended, diffuse NCI volumes whilst electrostatic interactions produce compact, localised regions. The charge‐to‐volume ratio $\frac{q_{\mathrm{NCI}}}{V_{\mathrm{NCI}}}$ emerges as a quantitative descriptor of this localisation continuum, with eigenvalue analysis through $\delta =\lambda_{2}/\lambda_{1}$ providing complementary directional information. This electron density‐based classification transcends traditional interaction nomenclature, offering systematic prediction of both structural stability and dynamic behaviour across diverse non‐covalent systems.

## Graphical abstract

Energy decomposition and electron density analyses reveal that hydrogen, pnictogen, and halogen bonds belong to a single continuum of non‐covalent interactions. The NCI charge‐to‐volume ratio quantitatively links electrostatic and dispersive contributions, providing a unified, density‐based description of interaction character across bonding families.

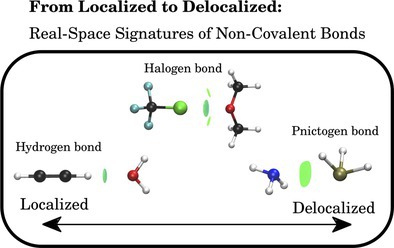

## 1 Introduction

Recently, non‐covalent interactions (NCIs) have been the subject of a vast number of studies from both theoretical and practical considerations. NCIs are of paramount importance in chemistry, bio‐disciplines and material sciences [1, 2, 3, 4, 5, 6, 7, 8, 9, 10, 11, 12]. Molecular clusters, the interaction of a protein and a drug, or the self‐assembly of nanomaterials, are mainly stabilised by NCIs [13, 14, 15]. This class of interactions spans a wide range of binding energies, and traditionally encompasses hydrogen bonding (HB), dipole–dipole interaction and London dispersion. Probably the most important difference between HB and the rest of the weak interactions was directionality [16]. Hydrogen bonding was usually characterised by being highly directional, in the sense that the electron donor and acceptor form an angle of almost 180°. This preference for a linear orientation has usually been considered a consequence of its directional nature [17], and it was taken as a unique feature among other NCIs, which did not show a marked orientation preference. However, the discovery of new interaction types has recently changed this panorama [18, 19].

With the advent of molecular beam and cryogenic experimental methods as well as the ever‐advancing theoretical methods, HBs have been proven to exist in $H_{2}S$ as well [20]. The elusive nature of hydrogen bonding and chemical bonding in general motivated the International Union of Pure and Applied Chemistry (IUPAC) to revisit its definition. The result of such a task was a set of guidelines to characterise hydrogen bonds not only from practical perspectives but also from theoretical considerations, considerably enlarging its original definition. In a nutshell, we may define a hydrogen bond X‐H ⋯ Y‐Z as an attractive interaction between a positively charged hydrogen and two electronegative species X and Y. Atom X is termed the proton donor and atom Y is called the proton acceptor. Traditionally, the role of Y has been undoubtedly assigned to O or N; however, less electronegative atoms, such as C, or even negatively charged regions, such as σ or π bonds, are now also accepted as proton acceptors [20]. Moreover, the new definition of HBs makes room for much less directional interactions (e.g., very weak HBs [21]). Overall, this entails a change in the paradigm of HB, where HBs do not involve two atoms (electron donor and acceptor) but instead can be more delocalised, involving a greater number of atoms.

To add to this change of paradigm, chemists have identified in the last decades a wealth of new bonding types along the periodic table. Halogen bonds (XBs) (group 17) are frequently exploited for crystal engineering [18], medicinal chemistry [22] and drug discovery [23]. Recently, similar bonding mechanisms have been proposed for adjacent main‐group elements, and non‐covalent “chalcogen bonds” (group 16) [24, 25] and “pnictogen bonds” (group 15) [19, 26, 27] have also been identified in crystal structures. Recently, even carbon bonding (group 14) [28] has been proposed as a stabilising interaction.

Some of these interactions break down the assumption that traditional HBs entail “special directional characteristics”. Indeed, the venue of halogen bonding broadened the spectrum of directional NCIs. In a halogen bond, the halogen atom plays the role of H in a hydrogen bond X‐Hal ⋯ Y‐Z, where Hal refers to an electropositive halogen atom and X, Y, and Z fulfil the same roles as they do in a hydrogen bond. Although with some exceptions, especially pnictogen bonding, the unexpected directionality of the great majority of the new non‐conventional NCIs has found a common origin; the σ‐hole concept [26].

In the coming sections, we briefly show some representative interactions along the periodic table, simultaneously highlighting the energetic terms and directional concept, to pinpoint how they set up a continuous scenario of NCIs.

## 2 Theoretical Background

### 2.1 NCI

The evaluation of non‐covalent interactions and their properties is challenging, especially when applying density functional theory (DFT). For instance, most of the current functionals fail to describe attractive dispersion interactions properly at large intermolecular distances. Moreover, the majority of density functionals cannot properly account for weak interactions at short and medium range [29], although this has been revised with the addition of empirical dispersion factors, for example, Grimme's D3 term [30, 31]. Although non‐covalent interactions are a pitfall for DFT, energetics and the density $\rho \left(r\right)$ enable the identification of weak interactions in molecules. Based on these concepts, the non‐covalent interaction index (NCI) [16] reveals and characterises weak interactions of various strengths according to the reduced density gradient, *s*(**r**).

(1) $$s\left(r\right)=\frac{|\nabla \rho \left(r\right)|}{2{\left(3\pi^{2}\right)}^{1/3}\rho {\left(r\right)}^{4/3}}$$

**FIGURE 1:**
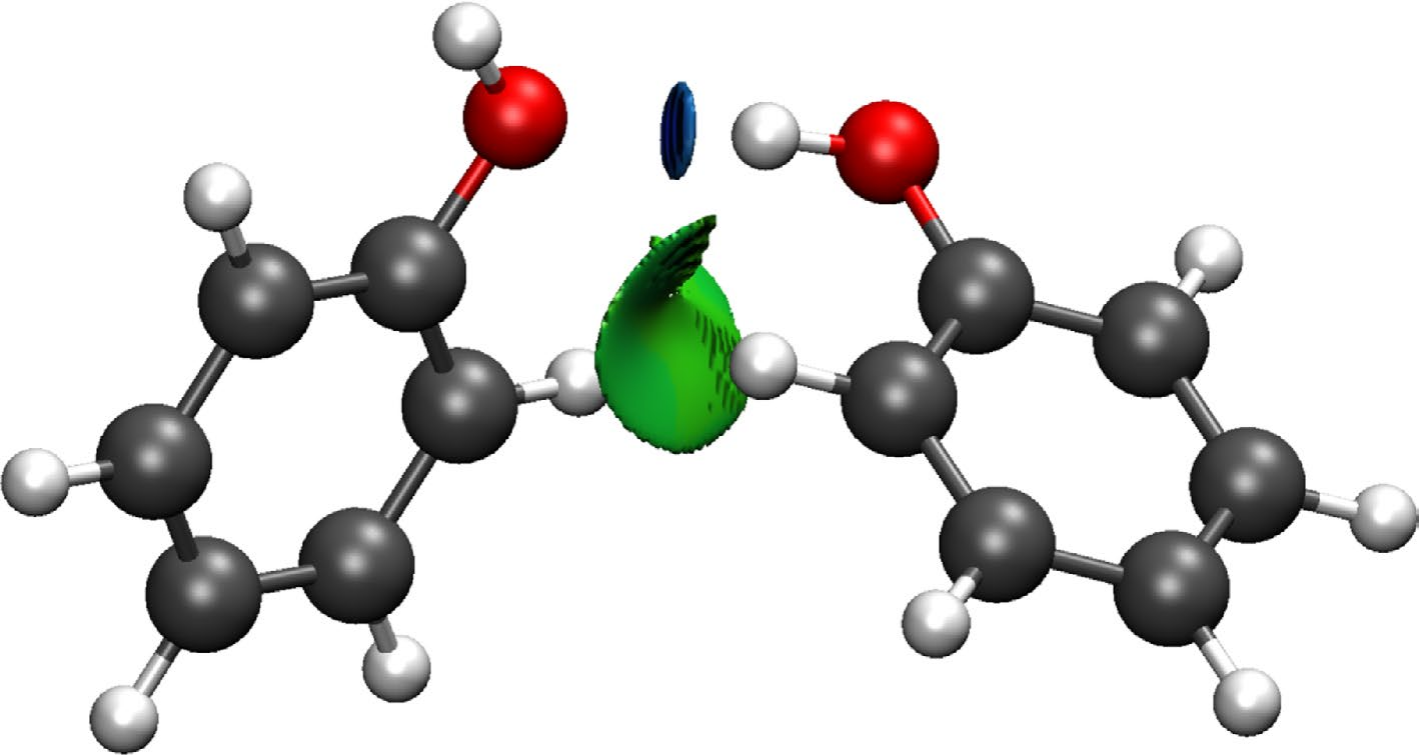
Phenol dimer with NCI surfaces (*s* = 0.5), colour range: $-0.03\leq \mathrm{sign}\left(\lambda_{2}\right)\rho \leq 0.03\;a.u$.

NCI unveils both stabilising (hydrogen bonds and van der Waals) and destabilising (steric clashes) interactions in real space in a very chemically intuitive manner. They are identified as those where both s and ρ are low. As illustrated in Figure 1, hydrogen bonds give rise to disk‐like isosurfaces (i.e., localised interactions mainly due to a two‐body interaction), whereas delocalised interactions appear as extended surfaces. It should be noted that this is related to the fact that in the Quantum Theory of Atoms in Molecules (QTAIM) [32, 33] chemical interactions often exhibit regions of low density gradient. However, NCI can detect very weak interactions that do not represent any electron density critical points [34, 35].

In a more recent interpretation of the reduced density gradient [34], it has been shown that the reduced density gradient is related to the bosonic kinetic energy density, $t_{\text{bose}}\left(r\right)$:

(2) $$t_{\text{bose}}\left(r\right)=\frac{t_{w}\left(r\right)}{t_{\mathrm{TF}}\left(r\right)}$$

where $t_{w}\left(r\right)=1/8{\left(\nabla \rho \left(r\right)\right)}^{2}/\rho \left(r\right)$ is the kinetic energy density for a bosonic system of density $\rho \left(r\right)$ or von Weizsäcker kinetic energy density; and $t_{\mathrm{TF}}\left(r\right)=3/10{\left(3\pi^{2}\right)}^{2/3}\rho {\left(r\right)}^{5/3}$ is the Thomas‐Fermi kinetic energy density for the homogeneous electron gas [36]. This reference enables the normalisation of the bosonic contribution, which otherwise is density dependent. $t_{\text{bose}}\left(r\right)$ constitutes the second term of the electron localisation function's kernel, $\chi \left(r\right)=t\left(r\right)/t_{\mathrm{TF}}\left(r\right)-t_{\text{bose}}\left(r\right)$, where $t\left(r\right)$ is the kinetic energy density [37]. $s\left(r\right)$ is directly related to $t_{\text{bose}}\left(r\right)=\frac{5}{3}s{\left(r\right)}^{2}$, so that non‐covalent regions revealed by the reduced density gradient correspond to regions of marked bosonic character [34].

This results in a subdivision into three categories according to the sign of the second eigenvalue $\lambda_{2}$ of the Hessian and the density ρ: strongly bonding (high ρ, $\lambda_{2}<0$, e.g., hydrogen bonds), very weak (low ρ, e.g., van der Waals) and repulsive steric clashes (high ρ, $\lambda_{2}>0$).

Integration of the density within the interaction region allows us to obtain the NCI charges and volumes ($V_{\mathrm{NCI}}\;\text{and}\;q_{\mathrm{NCI}}$), respectively (Equation 3 and 4). The integrated NCI volumes are defined uniquely according to best practices set out in Reference [21], which uses NCIPLOT with **s** = 1, $\gamma_{\mathrm{ref}}$ = 0.85, and intermolecular mode. Ranges within the NCI region can be assigned to a specific NCI interaction. Hence, three ranges with their respective NCI interaction volume and charge can be defined accordingly: the attractive ($V_{\mathrm{att}},q_{\mathrm{att}}$: −0.2 a.u. < ρ sign($\lambda_{2}$) < −0.02 a.u.), van der Waals ($V_{\mathrm{vdw}},q_{\mathrm{vdw}}$: −0.02 a.u. < ρ sign($\lambda_{2}$) < 0.02 a.u.) and repulsive region ($V_{\mathrm{rep}},q_{\mathrm{rep}}$: 0.02 a.u. < ρ sign($\lambda_{2}$) < 0.2 a.u.).

(3) $$V_{\mathrm{NCI}}=\int_{\Omega_{\mathrm{NCI}}}\;d\left(r\right)$$

(4) $$q_{\mathrm{NCI}}=\int_{\Omega_{\mathrm{NCI}}}\rho \left(r\right)\;d\left(r\right)$$

### 2.2 Energetic Partition

Within the ADF software, a Morokuma‐type energy decomposition method is implemented [38]. based on the Kohn‐Sham MO theory and, additionally, the fragment approach, the interaction can be decomposed by three different contributions or important physical terms as seen in Equation (5) [39].

(5) $$\Delta E_{\mathrm{int},\mathrm{EDA}}=\Delta V_{\text{elst}}+\Delta E_{\text{Pauli}}+\Delta E_{\mathrm{oi}}=\Delta E_{\text{steric}}+\Delta E_{\mathrm{oi}}$$

$\Delta V_{\text{elst}}$ represents the classical electrostatic interaction between unperturbed charge distributions of two fragments A and B, $\rho_{A}$ and $\rho_{B}$, respectively, resulting in an overall density ρ equal to $\rho_{A}$ + $\rho_{B}$. The antisymmetrised and renormalised wave function associated with this overall density gives rise to the Pauli repulsion ($\Delta E_{\text{Pauli}}$). This quantity is connected to destabilising interactions between occupied orbitals responsible for steric repulsion between the molecular fragments. Both $\Delta V_{\text{elst}}$ and $\Delta E_{\text{Pauli}}$ can be combined in a new term called the steric interaction, considering neutral fragments ($\Delta E_{\text{steric}}$) [40]. The orbital interaction energy is connected to charge transfer, polarisation and electron pair bonding. The final component is the dispersion contribution as obtained using Grimme's empirical dispersion correction [30], $E_{\text{disp}}$. $\Delta E_{\mathrm{int}}$ is the sum of $\Delta E_{\mathrm{int},\mathrm{EDA}}$ and $\Delta E_{\text{disp}}$ terms.

## 3 Computational Methods

All calculations were performed with Gaussian 09D rev01 [41] except for CCSD and MP2 calculations for chalcogen bonds, which were computed with Gaussian 16 due to size‐related convergence issues. We decided on MP2 for its well‐established accuracy in geometry optimisation of small molecular complexes where dispersion is an important factor [42]. Optimisation and frequency calculations were carried out at MP2/aug‐cc‐pVDZ [43, 44, 45, 46] level of theory using the Gaussian 09D package, except for iodine, where the pseudopotential def2‐TZVP was used. All the considered geometries correspond to minima on the potential energy surface. Interaction energies were computed as the difference between the dimer and the sum of the monomers having the same structure as in the complex, yielding $E_{\mathrm{int}}$. These quantities were also corrected for basis set superposition error by the counterpoise procedure, to which we will refer as $E_{\mathrm{int}}^{\mathrm{CP}}$ throughout the manuscript.

Bond energy analyses were executed with the ADF software at the PBE0/TZP level of theory, which was used to obtain EDA [47]. Psi4 was used to calculate the dispersion energies (D3 correction with the Becke‐Johnson damping function for the PBE0 functional) using the same geometries [30, 48, 49]. QTAIM analysis was performed by using AIMAll software [50] in order to obtain the density at the bond critical points and the three eigenvalues of the Hessian matrix. The NCI charges and volumes were obtained with NCIPLOT 4 [51, 52] at the promolecular level. In order to assure the convergence of NCI volumes ($V_{\mathrm{NCI}}$), a 0.1 Å grid step was used along each axis. The NCI isosurfaces were visualised with VMD version 1.9.2 [53].

## 4 Results

In this section, we will analyse density‐based and energetic descriptors across different families of non‐covalent interactions. We place particular emphasis on aspects of directionality and the spatial distribution of the interaction density.

### 4.1 Non‐Conventional Hydrogen Bonds

**FIGURE 2:**
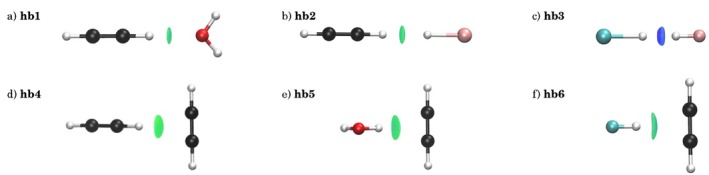
3D plots for hydrogen bonded complexes: (a) HCCH ⋯ ${\mathrm{OH}}_{2}$, (b) HCCH ⋯ HLi, (c) FH ⋯ HLi (d) HCCH $\cdots \left(\pi \right)$ HCCH, (e) HOH $\cdots \left(\pi \right)$ HCCH, (f) FH $\cdots \left(\pi \right)$ HCCH. NCI isosurfaces correspond to *s* = 0.5 and a colour scale of −0.04 < sign($\lambda_{2}$) ρ < +0.04 a.u.

Since non‐conventional hydrogen bonds represented a breakthrough in the definition of non‐covalent interactions, we will start by examining six weakly hydrogen‐bonded dimers that showcase a range of donor–acceptor motifs, including carbon‐based acceptors, carbon donors, and π‐type hydrogen bonds. Specifically, the studied systems comprise HCCH ⋯ ${\mathrm{OH}}_{2}$, HOH ⋯π, HCCH ⋯π, HCCH ⋯ HLi, FH ⋯π, and FH ⋯ HLi (see Figure 2). The π‐complexes (**hb4**–**hb6**) adopt T‐shaped geometries, where the acetylene π‐electrons function as proton acceptors. In contrast, the other dimers (**hb1**–**hb3**) adopt near‐linear arrangements in which carbon or fluorine acts as a proton donor, with lithium serving as a strong ionic acceptor where applicable.

The energy decomposition analysis for hydrogen‐bonded complexes revealed systematic variations in stabilisation mechanisms that correlate with both molecular geometry and the electronic properties of the participating species. In the π‐interacting systems (**hb4–6**), where the hydrogen bond donor interacts with the acceptor in a perpendicular orientation, the electrostatic attraction (${\mathrm{\Delta V}}_{\text{elst}}$ = −2.20 to −5.98 kcal/mol) was largely counterbalanced by Pauli repulsion ($\Delta E_{\text{Pauli}}$ = 2.08 to 5.73 kcal/mol), resulting in minimal net Coulombic contribution, as compiled in Table 1. Under these geometric constraints, the orbital interaction term ($\Delta E_{\mathrm{oi}}$ = −1.63 to −5.28 kcal/mol) emerges as the primary stabilising component, consistent with the charge‐transfer character inherent to π‐hydrogen bonding motifs. Conversely, the linear hydrogen‐bonded complexes **hb1** and **hb2** exhibit a classical electrostatic‐dominated interaction profile, where ${\mathrm{\Delta V}}_{\text{elst}}$ (−4.12 and −6.39 kcal/mol, respectively) provides the principal driving force for complexation. The enhanced electrostatic contribution in **hb2** relative to **hb1** reflects the greater charge separation in the Li‐H bond compared to the O‐H bond. The behaviour of **hb3** warrants particular attention: despite its linear geometry, the substantial orbital interaction component ($\Delta E_{\mathrm{oi}}$ = −16.09 kcal/mol) approaches the magnitude of the electrostatic term (${\mathrm{\Delta V}}_{\text{elst}}$ = −17.13 kcal/mol), suggesting significant covalent character relative to other studied complexes arising from the highly polarised F‐H and Li‐H bonds. This deviation from purely electrostatic behaviour in **hb3** indicates that bond polarity, rather than geometry alone, can drive orbital mixing and charge transfer even in linear hydrogen bonding arrangements.

**TABLE 1:** Energy decomposition analysis for the weakly bonded complexes at PBE0‐D3(BJ)/TZP level of theory.

Complex	ID	${\mathrm{\Delta V}}_{\text{elst}}$	$\Delta E_{\text{Pauli}}$	$\Delta E_{\mathrm{oi}}$	$\Delta E_{\text{disp}}$	$\Delta E_{\text{steric}}$	$\Delta E_{\mathrm{int}}$
$\text{HCCH}\cdots {\mathrm{OH}}_{2}$	**hb1**	−4.12	3.09	−2.01	−0.36	−1.10	−3.47
HCCH⋯HLi	**hb2**	−6.39	5.48	−4.12	−0.33	−0.90	−5.35
FH ⋯ HLi	**hb3**	−17.13	16.70	−16.09	−0.26	−0.43	−16.78
HOH ⋯π	**hb4**	−3.58	3.18	−2.52	−0.60	−0.40	−3.52
HCCH ⋯π	**hb5**	−2.20	2.08	−1.63	−0.64	−0.12	−2.39
FH ⋯π	**hb6**	−5.98	5.73	−5.28	−0.51	−0.19	−5.98
$H_{3}P\cdots {\mathrm{NH}}_{3}$	**p1**	−3.49	4.08	−1.76	−0.82	0.60	−1.98
${\mathrm{PH}}_{3}\cdots {\mathrm{NH}}_{3}$	**p2**	−1.98	2.16	−1.36	−0.46	0.17	−1.65
$H_{3}P\cdots {\mathrm{PH}}_{3}$	**p3**	−1.84	2.41	−1.53	−1.04	0.57	−2.00
${\mathrm{PH}}_{3}\cdots H_{3}P$	**p4**	−0.97	1.46	−0.81	−1.11	0.49	−1.43
$H_{3}N\cdots \mathrm{TMA}$	**p5**	−7.33	7.42	−3.64	−1.33	0.09	−4.88
$H_{3}P\cdots \mathrm{TMA}$	**p6**	−6.42	8.48	−3.56	−2.18	2.06	−3.68
$H_{3}\mathrm{As}\cdots \mathrm{TMA}$	**p7**	−9.06	12.42	−4.03	−2.51	3.36	−3.18
$H_{3}N\cdots \mathrm{TMP}$	**p8**	−3.22	3.09	−2.05	−1.09	−0.13	−3.27
$H_{3}P\cdots \mathrm{TMP}$	**p9**	−3.37	3.05	−2.01	−1.63	−0.32	−3.96
$H_{3}\mathrm{As}\cdots \mathrm{TMP}$	**p10**	−3.65	4.99	−2.19	−1.86	1.34	−2.71
${\mathrm{CF}}_{3}\mathrm{Cl}\;\cdots \mathrm{DME}$	**X1**	−3.63	3.39	−1.91	−1.16	−0.25	−3.32
${\mathrm{CF}}_{3}\mathrm{Cl}\cdots \mathrm{DMS}$	**X2**	−3.33	3.54	−2.43	−1.43	0.21	−3.65
${\mathrm{CF}}_{3}\mathrm{Br}\cdots \mathrm{DME}$	**X3**	−5.89	5.83	−3.15	−1.36	0.06	−4.45
${\mathrm{CF}}_{3}\mathrm{Br}\cdots \mathrm{TMA}$	**X4**	−12.18	13.94	−7.18	−2.00	1.76	−7.42
${\mathrm{CF}}_{3}\mathrm{Br}\cdots \mathrm{TMP}$	**X5**	−6.20	6.36	−4.31	−1.27	0.16	−5.42
${\mathrm{CF}}_{3}I\cdots \mathrm{TMA}$	**X6**	−19.88	24.14	−10.77	−2.63	4.26	−9.14
${\mathrm{CF}}_{3}I\cdots \mathrm{NHC}$	**X7**	−26.26	30.55	−14.46	−1.50	4.28	−11.68

*Note:* The total electrostatic interaction (${\mathrm{\Delta V}}_{\text{elst}}$), total Pauli repulsion (${\mathrm{\Delta E}}_{\text{Pauli}}$), total orbital interactions (${\mathrm{\Delta E}}_{\mathrm{oi}}$), dispersion energy (${\mathrm{\Delta E}}_{\text{disp}}$), steric repulsion (${\mathrm{\Delta E}}_{\text{steric}}$), and total interaction energy (${\mathrm{\Delta E}}_{\mathrm{int}}$) are all in kcal/mol.

The NCI analysis provides quantitative insight into the spatial distribution and electronic characteristics of hydrogen bonding interactions, revealing systematic trends that complement the EDA findings, which are systematised in Table 2. The integrated NCI charges ($q_{\mathrm{NCI}}$) for weak hydrogen bonds span a narrow range (0.09–0.33 a.u.), yet exhibit clear correlations with donor‐acceptor electronic properties. Linear hydrogen bonds involving highly electronegative atoms or ionic species (**hb2**: 0.12 a.u., **hb3**: 0.33 a.u.) demonstrate enhanced charge accumulation within the NCI region compared to the baseline acetylene‐water complex (**hb1**: 0.09 a.u.). The exceptional behaviour of **hb3** (FH ⋯ HLi), with the highest $q_{\mathrm{NCI}}$ value, reflects the synergistic effect of both highly polarised F‐H and Li‐H bonds, consistent with its substantial orbital interaction component observed in the EDA.

**TABLE 2:** NCI integrals of charge, volume, and vdW‐region‐only volume ($q_{\mathrm{NCI}}$, $V_{\mathrm{NCI}},V_{\mathrm{vdW}}$), alongside $\frac{q_{\mathrm{NCI}}}{V_{\mathrm{NCI}}}$ and $\frac{q_{\mathrm{NCI}}}{V_{\mathrm{vdW}}}$ for complexes discussed in text.

Complex	ID	$q_{\mathrm{NCI}}$	$V_{\mathrm{NCI}}$	$V_{\mathrm{vdW}}$	$\frac{q_{\mathrm{NCI}}}{V_{\mathrm{NCI}}}$	$\frac{q_{\mathrm{NCI}}}{V_{\mathrm{vdW}}}$	$\rho_{\mathrm{bcp}}\cdot 10^{3}$	$\delta =\frac{\lambda_{2}}{\lambda_{1}}$
HCCH ⋯ ${\mathrm{OH}}_{2}$	**hb1**	0.09	6.75	5.92	0.0137	0.0156	14.38	1.06
HCCH ⋯ HLi	**hb2**	0.12	8.29	7.10	0.0147	0.0171	14.21	1.00
FH ⋯ HLi	**hb3**	0.33	10.88	3.08	0.0299	0.1055	37.43	1.06
HOH ⋯π	**hb4**	0.23	15.99	13.21	0.0141	0.0171	12.24	1.50
HCCH ⋯π	**hb5**	0.16	14.28	13.69	0.0113	0.0118	8.18	1.37
FH ⋯π	**hb6**	0.30	14.55	7.45	0.0208	0.0406	37.43	1.00
$H_{3}P$ ⋯ ${\mathrm{NH}}_{3}$	**p1**	0.13	16.24	16.23	0.0083	0.0083	7.81	1.02
${\mathrm{PH}}_{3}\cdots {\mathrm{NH}}_{3}$	**p2**	0.06	7.27	7.27	0.0029	0.0082	9.17	1.01
$H_{3}P\cdots {\mathrm{PH}}_{3}$	**p3**	0.14	20.54	20.54	0.0187	0.0066	6.73	1.03
${\mathrm{PH}}_{3}\cdots H_{3}P$	**p4**	0.00	0.00	0.00	0.0000	0.0000	4.37	1.38
$H_{3}N\;\cdots \;\mathrm{TMA}$	**p5**	0.34	25.10	20.53	0.0135	0.0165	21.07	0.99
$H_{3}P\;\cdots \;\mathrm{TMA}$	**p6**	0.36	42.79	43.51	0.0082	0.0083	14.89	0.97
$H_{3}\mathrm{As}\;\cdots \;\mathrm{TMA}$	**p7**	0.46	52.28	50.37	0.0090	0.0087	17.83	0.99
$H_{3}N\;\cdots \;\mathrm{TMP}$	**p8**	0.19	21.37	21.25	0.0088	0.0088	10.82	0.91
$H_{3}P\;\cdots \;\mathrm{TMP}$	**p9**	0.18	28.27	28.27	0.0062	0.0062	8.64	0.95
$H_{3}\mathrm{As}\;\cdots \;\mathrm{TMP}$	**p10**	0.23	34.32	34.32	0.0067	0.0067	9.34	0.97
${\mathrm{CF}}_{3}\mathrm{Cl}\;\cdots \;\mathrm{DMA}$	**X1**	0.18	22.49	22.18	0.0081	0.0082	14.22	1.03
${\mathrm{CF}}_{3}\mathrm{Cl}\;\cdots \;\mathrm{DMS}$	**X2**	0.19	26.34	26.33	0.0071	0.0071	14.91	1.01
${\mathrm{CF}}_{3}\mathrm{Br}\;\cdots \;\mathrm{DME}$	**X3**	0.20	22.44	21.80	0.0087	0.0090	18.12	1.04
${\mathrm{CF}}_{3}\mathrm{Br}\;\cdots \;\mathrm{TMA}$	**X4**	0.35	32.04	28.15	0.0110	0.0126	27.32	1.00
${\mathrm{CF}}_{3}\mathrm{Br}\;\cdots \;\mathrm{TMP}$	**X5**	0.12	14.80	14.70	0.0081	0.0081	14.77	1.00
${\mathrm{CF}}_{3}I\;\cdots \;\mathrm{TMA}$	**X6**	0.57	44.73	36.60	0.0127	0.0155	36.93	1.00
${\mathrm{CF}}_{3}I\;\cdots \;\mathrm{NHC}$	**X7**	0.38	22.68	15.58	0.0168	0.0244	33.30	1.15

*Note:* QTAIM analysis measures of $\rho_{\mathrm{bcp}}$ and $\delta =\frac{\lambda_{2}}{\lambda_{1}}$ are also shown. All values are presented in atomic units, a.u. save for δ which is dimensionless.

In Figure 2, we can see that compounds **hb1–3** showed a small disk‐like NCI surface between the proton and the proton acceptor. Instead, for compounds **hb4–6**, this surface between the donor and the π bond was larger. However, it was difficult to distinguish the extent of localisation from the 3D isosurfaces alone. To go beyond this visual approach, it was possible to resort to the electron density derivatives in order to detect localisation quantitatively. As highlighted by Bohorquez et al. [54], the density eigenvalue at the bond critical point is a local tool that provides an understanding of the interaction shape. The electron density at the bond critical point ($\rho_{\mathrm{bcp}}$) revealed an apparent contradiction with energetic stability trends, see Table 2. The QTAIM analysis through electron density eigenvalues offered additional insight into interaction anisotropy. The eigenvalue ratio δ = $\lambda_{2}/\lambda_{1}$ serves as a quantitative descriptor of interaction directionality, with values approaching unity indicating isotropic, localised bonds. Linear hydrogen bonds (**hb1–3**) predominantly exhibited δ values near 1.0 (1.00–1.06), consistent with their directional character. Conversely, π‐hydrogen bonds showed greater deviation from unity (**hb4**: 1.50, **hb5**: 1.37), reflecting their inherently anisotropic nature due to π‐orbital involvement.

However, the metric presents limitations, as exemplified by **hb6** (FH ⋯π) showing δ = 1.00 despite its π‐character, suggesting that strong donor effects could override geometric considerations in determining eigenvalue behaviour. δ diverged from 1 as the multi‐atomic character of the bond emerges. Note that this information was usually used to distinguish π from σ covalent bonds [55].

The geometric differentiation between σ‐type and π‐type hydrogen bonds was manifested clearly in the NCI volume descriptors. π‐Hydrogen bonding systems (**hb4–6**) consistently exhibit larger interaction volumes ($V_{\mathrm{NCI}}$ = 14.28–15.99 a.u.) compared to their linear counterparts (**hb1–3**: 6.75–10.88 a.u.), reflecting the extended spatial distribution characteristic of π‐interactions, see Table 1. This volumetric expansion was seen for π‐system interactions and almost mirrored for $V_{\mathrm{NCI}}$, save for **hb3** and **hb6** where the division of volume was unevenly split, signifying a mixed interaction character.

The charge density descriptors $q_{\mathrm{NCI}}$, $V_{\mathrm{vdW}}$ and $\frac{q_{\mathrm{NCI}}}{V_{\mathrm{NCI}}}$ provided complementary measures of interaction intensity and localisation. π‐Hydrogen bonds consistently exhibited lower density ratios ($\frac{q_{\mathrm{NCI}}}{V_{\mathrm{NCI}}}$ = 0.0113–0.0299 a.u.) compared to linear systems. This trend reinforced the diffuse nature of π‐interactions, where electron density accumulation was distributed across a larger spatial region rather than concentrated at a specific donor‐acceptor contact point.

NCI integrations provided a robust framework for characterising hydrogen bond behaviour. π‐Hydrogen bonds consistently demonstrated larger interaction volumes coupled with lower charge densities, quantitatively confirming their delocalised character. The charge‐to‐volume ratios $\frac{q_{\mathrm{NCI}}}{V_{\mathrm{NCI}}}$ and $\frac{q_{\mathrm{NCI}}}{V_{\mathrm{vdW}}}$ served as particularly diagnostic descriptors, with π‐systems showing systematically lower values, reinforcing their dispersive rather than electrostatic character. The hydrogen‐bonded set exhibited a broad range of $\rho_{\mathrm{BCP}}$ values, from weak interactions around 8.18 × $10^{-3}$ a.u. to strong, localised bonding exceeding 37 × $10^{-3}$ a.u., reflecting diverse bonding motifs and matching the $\frac{q_{\mathrm{NCI}}}{V_{\mathrm{NCI}}}$ distribution. These findings establish a quantitative foundation for distinguishing hydrogen bond types and will serve as reference points for comparison with pnictogen and halogen bonding interactions.

### 4.2 Pnictogen Bonds

The pnictogen‐bonded complexes exhibited weak interaction energies (${\mathrm{\Delta E}}_{\mathrm{int}}$ = −1.43 to −4.88 kcal/mol) with diminished electrostatic contributions to the total (${\mathrm{\Delta V}}_{\text{elst}}$ = −0.97 to −9.06 kcal/mol) compared to hydrogen bonding systems. There are two subsets of pnictogen complexes: **p1–4** to investigate the effect of geometry and **p5–10** to explore the effect of element and interaction partner. For the first subset, complex (**p1**) showed the strongest electrostatic component (−3.49 kcal/mol) due to ammonia's enhanced basicity, whilst symmetric P–P interactions (**p3**, **p4**) displayed reduced electrostatic character (−1.84 and −0.97 kcal/mol) reflecting similar phosphorus electronegativity. Dispersion interactions provided crucial stabilisation (−0.46 to −1.11 kcal/mol), with enhanced contributions in phosphine‐phosphine systems (**p3**: −1.04 kcal/mol, **p4**: −1.11 kcal/mol) due to increased phosphorus polarisability. Notably, all pnictogen complexes exhibited positive steric repulsion terms (0.17–0.60 kcal/mol), contrasting with hydrogen bonding behaviour and indicating operation closer to the repulsive intermolecular potential wall. The pnictogen complexes furthermore contained a consistent orbital energy component (between −0.81 and −1.76 kcal/mol), which for complexes **p2–4** was comparable in size to the electrostatic term, therefore highlighting a significant contribution of the covalent character to the overall binding. This is consistent with previous publications dissecting energetic contributions to bonding [56, 57, 58]. For the second subset, depending on elemental makeup, the optimisation found the key σ‐hole donor‐acceptor interaction, which, due to stronger Lewis base partners, gave much stronger pnictogen bond energies. **p5** is an exception whereby the optimised geometry features an NH ⋯ N interaction. Nevertheless, we see a similar pattern in the binding, having significant contributions from all three, electrostatic, orbital, and dispersion energy terms, according to Table 1. All three terms were also increasing down the row (**p5** is the exception), showing more diversified contributors to binding for arsenic complexes. However, the disproportional growth in Pauli repulsion energy led to a decrease in overall binding energy for N to As complexes. Complexes with TMA (**p5–7**) were stronger than with TMP (**p8–10**), showcasing the importance of electrostatics in pnictogen interactions. This multifaceted stabilisation mechanism, balancing weak electrostatic, orbital, and dispersion contributions, underlaid the characteristically diffuse nature of pnictogen bonding.

**FIGURE 3:**
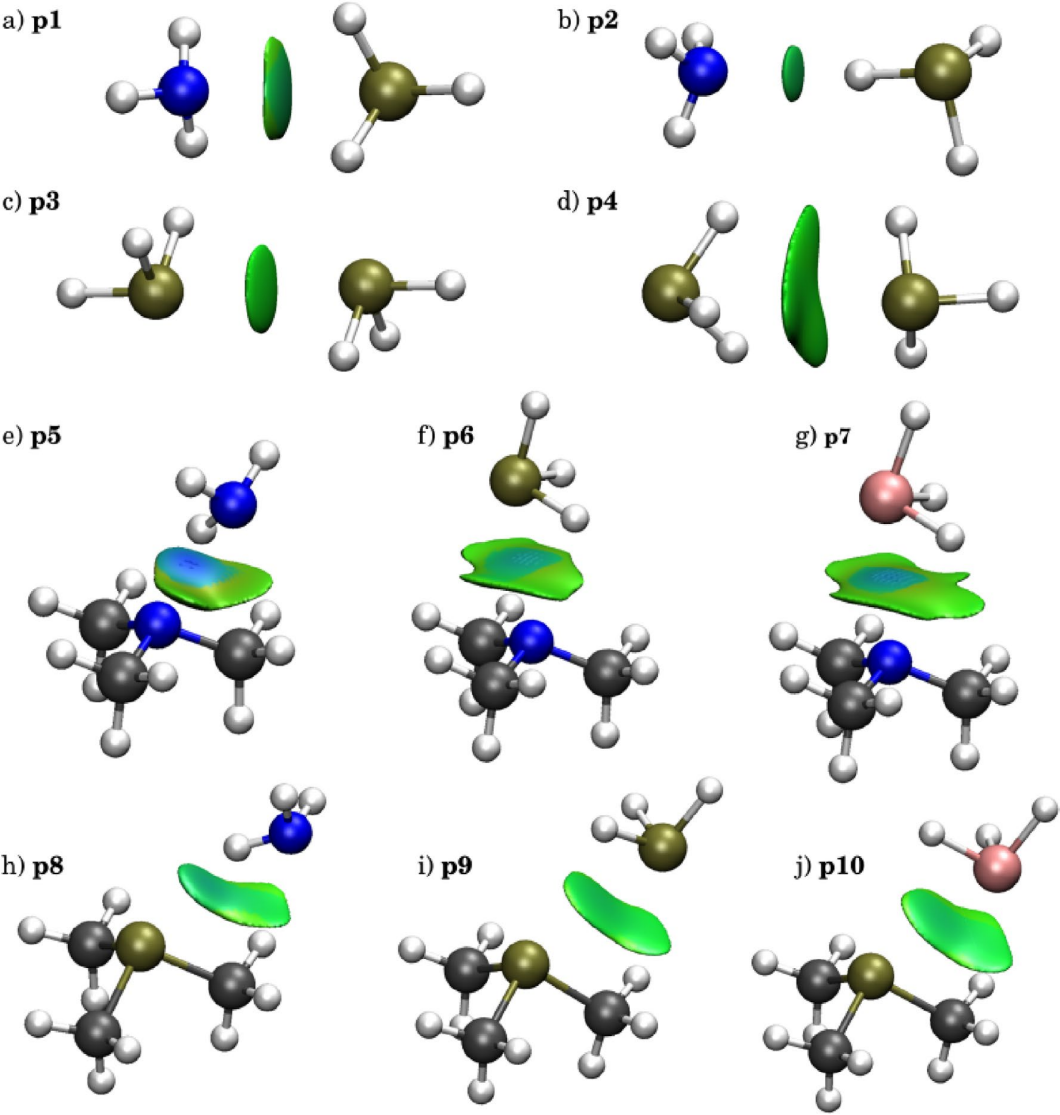
3D plots for pnictogen bond complexes: (a) $H_{3}$P ⋯ ${\mathrm{NH}}_{3}$, (b) ${\mathrm{PH}}_{3}\cdots H_{3}N$, (c) $H_{3}P\cdots {\mathrm{PH}}_{3}$, (d) ${\mathrm{PH}}_{3}\cdots H_{3}P$, $\left(e\right)\;{\mathrm{NH}}_{3}\;\cdots \;\mathrm{TMA},$ $\left(f\right)\;{\mathrm{PH}}_{3}\;\cdots \;\mathrm{TMA},$ $\left(g\right)\;{\mathrm{AsH}}_{3}\;\cdots \;\mathrm{TMA},$ $\left(h\right)\;{\mathrm{NH}}_{3}\;\cdots \;\mathrm{TMP},$ $\left(i\right)\;{\mathrm{PH}}_{3}\;\cdots \;\mathrm{TMP},$ $\left(j\right)\;{\mathrm{AsH}}_{3}\;\cdots \;\mathrm{TMP}$. NCI isosurfaces correspond to s = 0.5 and a colour scale of −0.04 < $\mathrm{sign}\left(\lambda_{2}\right)\rho <+0.04\;a.u$.

The NCI surfaces in Figure 3 revealed distinct interaction types within pnictogen‐bonded complexes, providing visual insight into the electronic distribution patterns. Complex **p2** displayed a small, localised disk characteristic of directional interactions, reflecting the concentrated electron density between the phosphine and ammonia. Complex **p5**'s surface in Figure 3e featured a dual nature of a stronger (bluer) localised interaction of NH ⋯ N alongside a diffuse section of the surface representing the CH ⋯ N interactions. In contrast, **p1** and **p3** exhibited more extended but still rounded surfaces, suggesting intermediate localisation with broader spatial distribution. Complexes **p4–10** showed flat surfaces indicative of delocalised dispersive interactions, where electron density was distributed across multiple atomic contacts rather than concentrated at a single interaction site. The electron density eigenvalues provided quantitative validation of these visual observations through the anisotropy parameter δ. Complex **p2** (Figure 3b) exhibited δ closest to unity (1.01), consistent with isotropic, localised bonding characteristic of directional interactions. Conversely, **p4** (Figure 3d) demonstrated the greatest deviation (δ = 1.38), reflecting the anisotropic nature of its dispersive character where electron density distribution lacked a preferred directionality. However, the remarkably similar δ values for **p1** (1.02) and **p3** (1.03) did not fully capture the morphological differences observed in their respective NCI surfaces. Complexes **p5** and **p7** also showed near‐unity, whereas the subsequent complexes' deviation from 1.0 reflected the delocalised interactions. **p7**'s relative isotropicity was also reflected in the blue colour concentration in its NCI region, see Figure 3g.

For complexes **p1–4**, the $\rho_{\mathrm{BCP}}$ values in the pnictogen series remained low overall—typically below 9 × 10^−3^ a.u., see Table 2, and they revealed a nuanced dependence on donor–acceptor asymmetry: interactions where the more electropositive phosphorus acted as the donor **p1** showed slightly higher BCP densities than their reversed counterparts **p4**, underscoring the directional sensitivity and weakly covalent nature of pnictogen bonding. Complexes **p5–10** had larger $\rho_{\mathrm{BCP}}$ values due to the use of stronger binding partners. They featured a key pattern of larger values for N and As complexes than for P; and similarly with P as TMP acceptor, the interactions were weaker than with N in TMA.

The van der Waals volume, $V_{\mathrm{NCI}}$, analysis revealed a clear progression that correlates with the interaction mechanism according to Table 2. Complex **p2** demonstrated the smallest volume (7.27 a.u.), reflecting its directional hydrogen bond character where interaction density was concentrated along the N–H ⋯ P axis. Complexes **p1** and **p3** exhibited substantially larger intermediate volumes (16.23 and 20.54 a.u., respectively), indicating expanded interaction regions where both electrostatic and dispersive contributions operated across multiple atomic contacts. Complex **p4** presented negligible integrated values, consistent with its highly delocalised nature, where conventional integration boundaries failed to capture the extended dispersive network.

For complexes **p1–4**, the low NCI charges and thus reduced charge accumulation reflected the inherently weaker electrostatic character of pnictogen interactions, where the lower electronegativity of phosphorus compared to oxygen or nitrogen results in less polarised donor‐acceptor pairs. The charge‐to‐volume ratios $\frac{q_{\mathrm{NCI}}}{V_{\mathrm{NCI}}}$ and $\frac{q_{\mathrm{NCI}}}{V_{\mathrm{vdW}}}$ further emphasised this trend, with values ranging from 0.0000 to 0.0187 a.u., significantly lower than those observed in hydrogen bonding systems, both compiled in Table 2. Complexes **p5–7** showed much increased $q_{\mathrm{NCI}}$, but the systematically increasing $V_{\mathrm{NCI}}$ kept the $\frac{q_{\mathrm{NCI}}}{V_{\mathrm{vdW}}}$ low. The pattern was repeated for **p8–10**, although with lower NCI charges and volumes, suggesting a worse density overlap between the interaction partners. Nevertheless, all complexes **p5–10** showed non‐zero NCI charges and low $\frac{q_{\mathrm{NCI}}}{V_{\mathrm{vdW}}}$ indicative of both non‐negligible electrostatic interaction, but also significant non‐polar character.

Based on these complementary analyses, pnictogen complexes could be systematically categorised into three distinct interaction regimes: highly localised interactions exemplified by **p2** and **p5**, where directional forces dominate; moderately localised interactions represented by **p1**, **p3**, and **p7**, where electrostatic and dispersive contributions operate cooperatively across expanded regions; and delocalised dispersive interactions typified by **p4**, **p6**, and **p8–10**, where stabilisation arises from cumulative weak interactions distributed across the molecular framework. This classification reflects the progressive transition from directional to dispersive bonding mechanisms within pnictogen systems, providing a framework for understanding the diverse nature of these interactions.

### 4.3 Halogen Bonds

Halogen‐bonded complexes demonstrated interactions where substantial stabilisation could be achieved through the cumulative effect of multiple local interactions. The selected family of ${\mathrm{CF}}_{3}X$ compounds (X = Cl, Br, I) paired with diverse acceptors (DME, DMS, TMA, TMP, and NHC) provided systematic variation in interaction strength and electronic character, encompassing the range of halogen bonding behaviour reported in the literature [59, 60, 61]. The energy decomposition analysis revealed significantly enhanced stability compared to pnictogen systems, with total interaction energies ranging from −3.32 to −11.68 kcal/mol, see Table 1. The electrostatic term was the largest single contributor to binding for all halogen bonds, rising most sharply down Group 17. For the chlorine and bromine complexes (**X1–6**), the electrostatic attraction ($\Delta V_{\text{elst}}$ = −3.33 to −12.18 kcal/mol) was largely counterbalanced by Pauli repulsion ($\Delta E_{\text{Pauli}}$ = 3.39 to 13.94 kcal/mol), and for iodine complexes (**X6–7**), it even exceeded their electrostatic counterparts. The orbital interaction energy emerged as a large stabilising component across all halogen‐bonded systems, with contributions ranging from −1.91 to −14.46 kcal/mol. This trend was particularly pronounced in the iodine complexes (**X6**, **X7**), with substantial orbital interaction terms (−10.77 and −14.46 kcal/mol) reflecting the enhanced polarisability of heavier halogens and their capacity for charge transfer interactions, consistent with the established σ‐hole concept in halogen bonding which fundamentally describes halogen bond directionality through the anisotropic distribution of electron density that creates regions of positive electrostatic potential, rather than through charge transfer mechanisms [61]. Dispersion contributions also exhibit a proportional relationship with halogen size, increasing from −1.16 kcal/mol in **X1** to −2.63 kcal/mol in **X6**, contributing relatively less stabilisation upon descending the group.

Three distinct interaction regimes emerged from this analysis: chlorine‐containing complexes represent relatively weak interactions where dispersion maintains significant relevance alongside modest electrostatic and orbital contributions; iodine systems exhibit highly directional character dominated by electrostatic and orbital interactions; bromine complexes occupy an intermediate position where substantial compensation between electrostatic attraction and Pauli repulsion occurs, with orbital interactions providing the decisive stabilisation. This progression illustrates the transition from dispersion‐augmented interactions to charge‐transfer‐dominated bonding as halogen polarisability increased.

The halogen‐bonded complexes also exhibit systematic trends in NCI descriptors that correlate directly with halogen identity and acceptor characteristics, according to Table 2. Moving from chlorine to iodine produces a pronounced increase in both integrated NCI charge ($q_{\mathrm{NCI}}$: 0.18 to 0.57 a.u.) and interaction volume ($V_{\mathrm{NCI}}$: 22.49 to 44.73 a.u.), reflecting the enhanced polarisability and σ‐hole strength of heavier halogens. This progression indicates increasingly localised and stronger halogen bonds down the periodic group, with iodine complexes demonstrating the most concentrated charge distributions.

**FIGURE 4:**
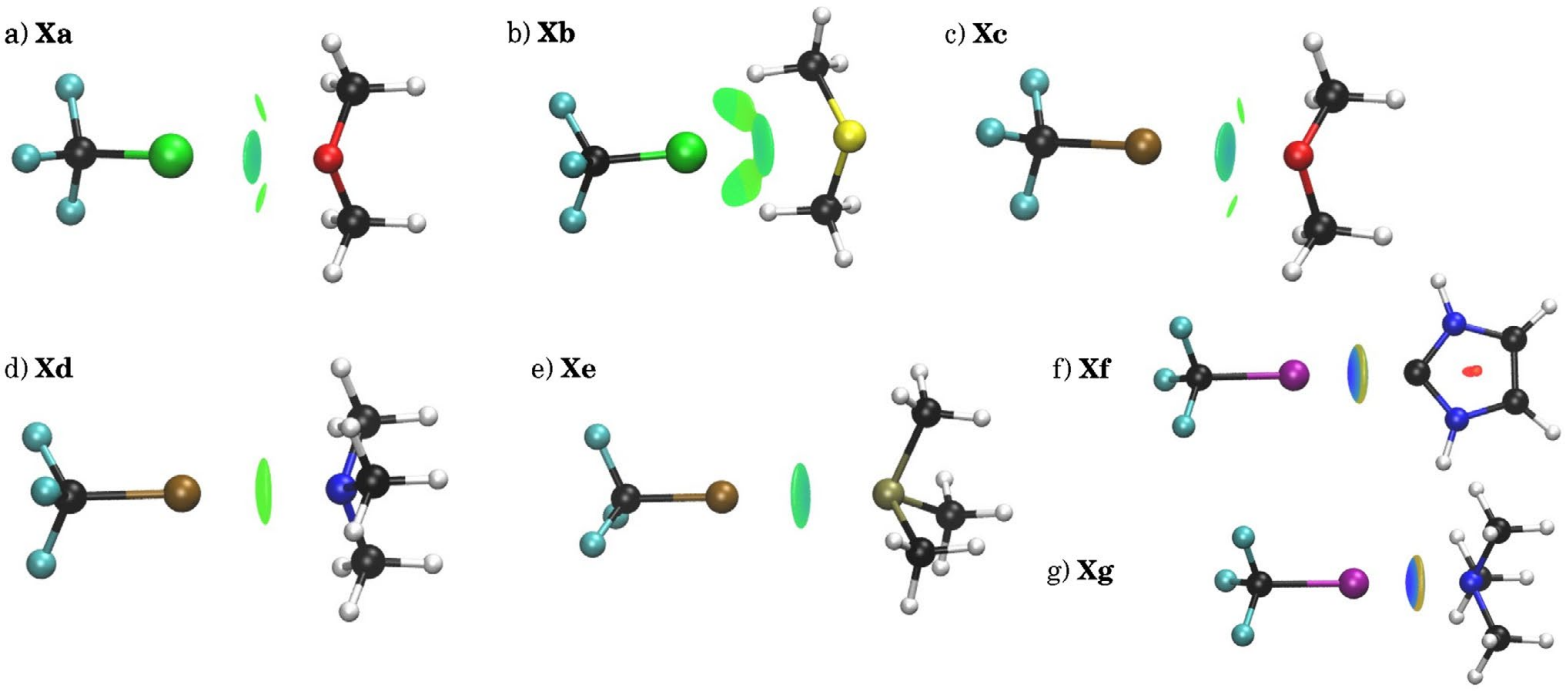
3D plots for halogen bond complexes (left to right): (a) $\;{\mathrm{CF}}_{3}\mathrm{Cl}\cdots \mathrm{DME},$ (b) $\;{\mathrm{CF}}_{3}\mathrm{Cl}\cdots \mathrm{DMS},$ $\left(c\right)\;{\mathrm{CF}}_{3}\mathrm{Br}\cdots \mathrm{DME},$ $\left(d\right)\;{\mathrm{CF}}_{3}\mathrm{Br}\cdots \mathrm{TMA},$ $\left(e\right)\;{\mathrm{CF}}_{3}\mathrm{Br}\cdots \mathrm{TMP},$ $\left(f\right)\;{\mathrm{CF}}_{3}I\cdots \mathrm{NHC},$ $\left(g\right)\;{\mathrm{CF}}_{3}I\cdots \mathrm{TMA}$. NCI isosurfaces correspond to s = 0.5 and a colour scale of $-0.04<\mathrm{sign}\left(\lambda_{2}\right)\rho <+0.04\;a.u$.

The acceptor identity significantly modulated interaction characteristics, with nitrogen‐containing acceptors (TMA, TMP) producing more localised and directional isosurfaces characterised by δ values approaching unity. These flat, disk‐like NCI surfaces from Figure 4 confirmed strong electrostatic interactions, as exemplified by **X4** and **X6** (δ = 1.00) in Table 2. Conversely, oxygen acceptors (DME, DMS) generated less localised interactions with multi‐component isosurfaces, where individual NCI regions became increasingly separated as halogen size increased due to growing electrostatic contributions.

The electron density at bond critical points ($\rho_{\mathrm{bcp}}$) increased systematically with halogen atomic number (**X1**: 14.22 to **X6**: 36.93 × 10^−3^ a.u.), with the most significant variation occurring between chlorine and bromine systems. This trend reflected the enhanced covalent character in heavier halogen bonds, consistent with their increased orbital interaction components observed in the EDA analysis.

Volume partitioning revealed that van der Waals interactions dominated the total NCI volume across all halogen complexes, with the notable exception of **X7**, where attractive and dispersive regions contributed nearly equally ($V_{\mathrm{NCI}}$ = 22.68 a.u., $V_{\mathrm{vdW}}$ = 15.58 a.u.). The attractive volume ($V_{\mathrm{NCI}}–V_{\mathrm{vdW}}$) increased substantially down the periodic table, highlighting enhanced localisation for heavier halogens and supporting the transition from dispersion‐dominated to electrostatic‐controlled interactions.

The charge‐to‐volume ratios $\frac{q_{\mathrm{NCI}}}{V_{\mathrm{NCI}}}$ and $\frac{q_{\mathrm{NCI}}}{V_{\mathrm{vdW}}}$ provided the most discriminating descriptor of halogen bond character. Chlorine complexes exhibited low ratios (0.0071–0.0082 a.u.), confirming their delocalised, dispersion‐dominated nature. Iodine systems demonstrated substantially higher ratios (0.0155–0.0244 a.u.), indicating localised, electrostatic character. Bromine complexes showed intermediate behaviour with considerable variation (0.0081–0.0126), reflecting their transitional position between dispersive and electrostatic regimes.

This analysis revealed a continuous transition from delocalised, dispersion‐controlled interactions in chlorine systems to localised, electrostatically‐driven charge‐transfer bonding in iodine complexes, with bromine exhibiting intermediate characteristics. The charge‐based classification aligned with QTAIM trends and should be favoured over volume‐based categorisation, though the discrepancy between these approaches underscored the complex, intermediate nature of halogen bonding between purely electrostatic and dispersive interaction types.

## 5 Conclusion

This study has examined the energetic and electron density characteristics of non‐conventional hydrogen, pnictogen and halogen bonds, revealing systematic patterns that transcend traditional interaction classifications which exist along a continuum defined by the relative contributions of electrostatic, orbital, and dispersive components. EDA established clear mechanistic distinctions across interaction families, where linear hydrogen‐bonded complexes were predominantly driven by electrostatics, whereas other hydrogen bonds also exhibited significant orbital and dispersion contributions. Pnictogen bonding systems contained a mixed electrostatic, orbital, and dispersion contributions reflecting the polarity of the interacting elements, and producing an overall interaction more diffuse than found for other classes. The lighter halogen bonds demonstrated dispersion‐dominated interactions with relatively large $\Delta E_{\text{disp}}$ contributions to overall binding energy; however, electrostatic and orbital terms remained the biggest in value. Moving down the group to iodine complexes, the electrostatic terms showed the biggest increase, reflecting the increased importance of the σ‐hole in interactions.

The NCI analysis provides a unifying framework for characterising these diverse interactions. The NCI isosurfaces corresponding to dispersion‐driven interactions were large and flat, with near‐zero density integrals, reflecting their inherently diffuse character as observed in π‐hydrogen, pnictogen, and chlorine halogen bonds. Conversely, polar interactions, as well as those originating from charge transfer, showed up as small, compact NCI regions with larger NCI charge integrals. This inverse relationship between electrostatic contribution and $V_{\mathrm{NCI}}$ was quantitatively captured by the $\frac{q_{\mathrm{NCI}}}{V_{\mathrm{vdW}}}$ ratio, which increased systematically as interactions transition from dispersive to electrostatic character. The eigenvalue analysis (with δ) additionally showcased the low anisotropies of localised polar bonding and high anisotropies for more dispersive, multi‐centre bonds.

These findings support a unified classification scheme based on electron density topology, where interaction character is determined by the balance between localised electrostatic and delocalised dispersive forces. Electrostatic interactions generate compact, directional NCI regions concentrated around bond critical points, whilst dispersive interactions produce extended, anisotropic regions. This density‐based approach transcends traditional interaction categories, providing a semi‐quantitative framework for understanding the continuum of non‐covalent bonding behaviour across diverse chemical systems, stepping outside of the naming criteria based primarily on elements, and emphasising instead the physical properties governing the interactions.

## Conflicts of Interest

The authors declare no conflicts of interest.

## Supporting information


**Data S1:** Supplementary Information.

## Data Availability

The data that support the findings of this study are available in the Supporting Information of this article.

## References

[1] J. Cerny and P. Hobza , “Non‐covalent interactions in biomacromolecules,” Physical Chemistry Chemical Physics 9, no. 39 (2007): 5291–5303. 17914464 10.1039/b704781a

[2] J. Guo , C. Tian , and B. Xu , “Biomaterials based on noncovalent interactions of small molecules,” EXCLI Journal 20 (2020): 1124–1140. 10.17179/excli2020-2656 PMC7573174 33088250

[3] A. Haque , K. M. Alenezi , M. S. Khan , W.‐Y. Wong , and P. R. Raithby , “Non‐covalent interactions (ncis) in π‐conjugated functional materials: advances and perspectives,” Chemical Society Reviews 52, no. 2 (2023): 454–472. 36594823 10.1039/d2cs00262k

[4] P. Hobza and J. Rezac , “Introduction: Noncovalent interactions,” Chemical Reviews 116, no. 9 (2016): 4911–4912. 27166734 10.1021/acs.chemrev.6b00247

[5] S. Jena , J. Dutta , K. D. Tulsiyan , A. K. Sahu , S. S. Choudhury , and H. S. Biswal , “Noncovalent interactions in proteins and nucleic acids: beyond hydrogen bonding and π‐stacking,” Chemical Society Reviews 51, no. 11 (2022): 4261–4286. 35560317 10.1039/d2cs00133k

[6] D. Jovanovic , M. Poliyodath Mohanan , and S. M. Huber , “Halogen, chalcogen, pnictogen, and tetrel bonding in non‐covalent organocatalysis: An update,” Angewandte Chemie International Edition 63, no. 31 (2024): e202404823. 38728623 10.1002/anie.202404823

[7] T. Kim , G. Shin , T. Park , and M. Kim , “Molecular design leveraging non‐covalent interactions for efficient light‐emitting organic small molecules,” Advanced Functional Materials 35, no. 2 (2025): 2,412,267.

[8] Z. Li , R. Yu , and B. Guo , “Shape‐memory and self‐healing polymers based on dynamic covalent bonds and dynamic noncovalent interactions: Synthesis, mechanism, and application,” ACS Applied Bio Materials 4, no. 8 (2021): 5926–5943. 10.1021/acsabm.1c00606 35006922

[9] C. C. J. Loh , “Exploiting non‐covalent interactions in selective carbohydrate synthesis,” Nature Reviews Chemistry 5, no. 11 (2021): 792–815. 37117666 10.1038/s41570-021-00324-y

[10] X. Tang , J. Pang , J. Dong , Y. Liu , X. Bu , and Y. Cui , “Supramolecular assembly frameworks (safs): Shaping the future of functional materials,” Angewandte Chemie 136, no. 33 (2024): e202406956. 10.1002/anie.202406956 38713527

[11] J. Zhan , Z. Lei , and Y. Zhang , “Non‐covalent interactions of graphene surface: Mechanisms and applications,” Chem 8, no. 4 (2022): 947–979.

[12] Y. Zhang , X. Lin , Y. Wang , et al., “The non‐covalent and covalent interactions of whey proteins and saccharides: influencing factor and utilization in food,” Critical Reviews in Food Science and Nutrition 65, no. 20 (2025): 3896–3910. 38961829 10.1080/10408398.2024.2373386

[13] Y. Long , J. F. Hui , P. P. Wang , et al., “Hydrogen bond nanoscale networks showing switchable transport performance,” Scientific Reports 2 (2012): 1–6. 10.1038/srep00612 PMC3430880 22937221

[14] G. Sliwowski , K. Sandeepkumar , M. Jens , and E. W. Lowe, Jr. , “Computational methods in drug discovery,” Pharmacology Review 1 (2014): 334–395. 10.1124/pr.112.007336 PMC3880464 24381236

[15] S. Song , L. Wang , J. Su , et al., “Manifold dynamic non‐covalent interactions for steering molecular assembly and cyclization,” Chemical Science 12, no. 35 (2021): 11,659–11,667. 10.1039/d1sc03733a PMC8442717 34667560

[16] E. R. Johnson , S. Keinan , P. Mori‐Sánchez , J. Contreras‐García , A. J. Cohen , and W. Yang , “Revealing noncovalent interactions,” Journal of the American Chemical Society 132, no. 18 (2010): 6498–6506. 20394428 10.1021/ja100936w PMC2864795

[17] A. Martín Pendás , A. M. Blanco , and E. Francisco , “The nature of the hydrogen bond: A synthesis from the interacting quatum atoms picture,” Journal of Chemical Physics 125, no. 18 (2006): 184,112. 10.1063/1.2378807 17115743

[18] P. Metrangolo and G. Resnati , “Halogen bonding: A paradigm in supramolecular chemistry,” Chemistry—a European Journal 7, no. 12 (2001): 2511–2519. 11465442 10.1002/1521-3765(20010618)7:12<2511::aid-chem25110>3.0.co;2-t

[19] S. Scheiner , “The pnicogen bond: Its relation to hydrogen, halogen, and other noncovalent bonds,” Accounts of Chemical Research 46, no. 2 (2012): 280–288. 23135342 10.1021/ar3001316

[20] G. R. Desiraju , “A bond by any other name,” Angewandte Chemie International Edition in English 50, no. 1 (2011): 52–59. 10.1002/anie.201002960 21031379

[21] T. Kawase , K. Tanaka , N. Fujiwara , H. R. Darabi , and M. Oda , “Complexation of a carbon nanoring with fullerenes,” Angewandte Chemie International Edition in English 115, no. 114 (2003): 1662–1666. 10.1002/anie.200250728 12698460

[22] H. Matter , M. Nazaré , S. Güssregen , et al., “Evidence for C–Cl/C–Br … π interactions as an important contribution to protein–ligand binding affinity,” Angewandte Chemie International Edition in English 121, no. 16 (2009): 2955–2960. 10.1002/anie.200806219 19294721

[23] L. A. Hardegger , B. Kuhn , B. Spinnler , et al., “Systematic investigation of halogen bonding in protein–ligand interactions,” Angewandte Chemie International Edition in English 50, no. 1 (2011): 314–318. 10.1002/anie.201006781 21184410

[24] D. Manna and G. Mugesh , “Regioselective deiodination of thyroxine by iodothyronine deiodinase mimics: An unusual mechanistic pathway involving cooperative chalcogen and halogen bonding,” Journal of the American Chemical Society 134, no. 9 (2012): 4269–4279. 22352472 10.1021/ja210478k

[25] J. S. Murray , L. Pat , and P. Politzer , “A predicted new type of directional noncovalent interaction,” International Journal of Quantum Chemistry 107, no. 12 (2007): 2286–2292.

[26] G. Resnati , D. L. Bryce , G. R. Desiraju , et al., “Definition of the pnictogen bond (iupac recommendations 2023),” Pure and Applied Chemistry 96, no. 1 (2024): 135–145.

[27] S. Scheiner , “A new noncovalent force: Comparison of p ⋯ n interaction with hydrogen and halogen bonds,” Journal of Chemical Physics 134, no. 9 (2011): 094315. 21384977 10.1063/1.3562209

[28] D. Mani and E. Arunan , “The x‐c ⋯ y (x = o/f, y = o/s/f/cl/br/n/p) ‘carbon bond’ and hydrophobic interactions,” Physical Chemistry Chemical Physics 15 (2013): 14,377–14,383. 10.1039/c3cp51658j 23896956

[29] C. Corminboeuf , “Minimizing density functional failures for non‐covalent interactions beyond van der waals complexes,” Accounts of Chemical Research 47, no. 11 (2014): 3217–3224. 24655016 10.1021/ar400303a

[30] S. Grimme , J. Antony , S. Ehrlich , and H. Krieg , “A consistent and accurate ab initio parametrization of density functional dispersion correction (dft‐d) for the 94 elements h‐pu,” Journal of Chemical Physics 132, no. 15 (2010): 154,104. 10.1063/1.3382344 20423165

[31] S. Grimme , J. Antony , S. Ehrlich , and H. Krieg , “A consistent and accurate *ab initio* parametrization of density functional dispersion correction(dft‐d) for the 94 elements h‐pu,” Journal of Chemical Physics 15, no. 132 (2010): 154,104. 10.1063/1.3382344 20423165

[32] R. F. Bader , “A quantum theory of molecular structure and its applications,” Chemical Reviews 91, no. 5 (1991): 893–928.

[33] C. F. Matta and R. J. Boyd , An introduction to the quantum theory of atoms in molecules: The Quantum Theory of Atoms in Molecules: From Solid State to DNA and Drug Design (Wiley Online Library, 2007).

[34] R. A. Boto , J. Contreras‐García , J. Tierny , and J.‐P. Piquemal , “Interpretation of the reduced density gradient,” Molecular Physics 114, no. 7–8 (2016): 1406–1414.

[35] J. R. Lane , J. Contreras‐García , J.‐P. Piquemal , B. J. Miller , and H. G. Kjaergaard , “Are bond critical points really critical for hydrogen bonding?,” Journal of Chemical Theory and Computation 9, no. 8 (2013): 3263–3266. 26584086 10.1021/ct400420r

[36] A. D. Becke and K. E. Edgecombe , “A simple measure of electron localization in atomic and molecular systems,” Journal of Chemical Physics 92 (1990): 5397.

[37] A. Savin , O. Jepsen , J. R. Flad , O. K. Andersen , H. Preuss , and H. G. von Schnering , “Electron localization in solid‐state structures of the elements: the diamond structure,” Angewandte Chemie International Edition in English 31, no. 2 (1992): 187.

[38] K. Kitaura and K. Morokuma , “A new energy decomposition scheme for molecular interactions within the hartree‐fock approximation,” International Journal of Quantum Chemistry 10, no. 2 (1976): 325–340.

[39] T. Ziegler and A. Rauk , “On the calculation of bonding energies by the hartree‐fock slater method,” Theorefic Chimica Acta 46 (1977): 1–10.

[40] F. M. Bickelhaupt and E. J. Baerends , “Kohn‐sham density functional theory: predicting and understanding chemistry,” Reviews in computational chemistry 15 (2000): 1–86.

[41] M. Frisch , G. Trucks , H. Schlegel , et al., Gaussian 09 D. 01, Gaussian (Gaussian Inc., 2009).

[42] C. Puzzarini , “Accurate molecular structures of small‐ and medium‐sized molecules,” International Journal of Quantum Chemistry 116, no. 21 (2016): 1513–1519.

[43] T. H. Dunning, Jr. , “Gaussian basis sets for use in correlated molecular calculations. i. the atoms boron through neon and hydrogen,” Journal of Chemical Physics 90, no. 2 (1989): 1007–1023.

[44] R. A. Kendall , T. H. Dunning, Jr. , and R. J. Harrison , “Electron affinities of the first‐row atoms revisited. systematic basis sets and wave functions,” Journal of Chemical Physics 96, no. 9 (1992): 6796–6806.

[45] K. A. Peterson , D. E. Woon , and T. H. Dunning, Jr. , “Benchmark calculations with correlated molecular wave functions. iv. the classical barrier height of the h + h2 → h2+ h reaction,” Journal of Chemical Physics 100, no. 10 (1994): 7410–7415.

[46] D. E. Woon and T. H. Dunning, Jr. , “Gaussian basis sets for use in correlated molecular calculations. iii. the atoms aluminum through argon,” Journal of Chemical Physics 98, no. 2 (1993): 1358–1371.

[47] G. te Velde , F. M. Bickelhaupt , E. J. Baerends , et al., “Chemistry with adf,” Journal of Computuational Chemistry 22, no. 9 (2001): 931–967.

[48] S. Grimme , S. Ehrlich , and L. Goerigk , “Effect of the damping function in dispersion corrected density functional theory,” Journal of Computational Chemistry 32, no. 7 (2011): 1456–1465. 21370243 10.1002/jcc.21759

[49] D. G. A. Smith , L. A. Burns , A. C. Simmonett , et al., “Psi4 1.4: Open‐source software for high‐throughput quantum chemistry,” Chemical Physics 152 (2020): 184,108. 10.1063/5.0006002 PMC7228781 32414239

[50] C. F. Matta and R. J. Boyd , The Quantum Theory of Atoms in Molecules (Wiley‐VCH, 2007).

[51] R. Boto , F. Peccati , R. Laplaza , et al., “Nciplot4: A new step towards a fast quantification of noncovalent interactions,” Journal of Chemical Theory and Computation 16 (2020): 4150–4158. 32470306 10.1021/acs.jctc.0c00063

[52] J. Contreras‐García , W. Yang , and E. R. Johnson , “Analysis of hydrogen‐bond interaction potentials from the electron density: integration of noncovalent interaction regions,” Journal of Physical Chemistry A 115, no. 45 (2011): 12,983. 10.1021/jp204278k PMC3651877 21786796

[53] W. Humphrey , A. Dalke , and K. Schulten , “VMD—Visual Molecular Dynamics,” Journal of Molecular Graphics 14 (1996): 33–39. 8744570 10.1016/0263-7855(96)00018-5

[54] H. J. Bohórquez , C. F. Matta , and R. J. Boyd , “The localized electrons detector as an ab initio representation of molecular structures,” International Journal of Quantum Chemistry 110, no. 13 (2010): 2418–2425.

[55] R. F. W. Bader , T. S. Slee , D. Cremer , and E. Kraka , “Description of conjugation and hyperconjugation in terms of electron distributions,” Journal of the American Chemical Society 105, no. 15 (1983): 5061–5068.

[56] L. de Azevedo Santos , T. A. Hamlin , T. C. Ramalho , and F. M. Bickelhaupt , “The pnictogen bond: a quantitative molecular orbital picture,” Physical Chemistry Chemical Physics 23, no. 25 (2021): 13,842–13,852. 10.1039/d1cp01571k PMC8297534 34155488

[57] R. Liu , Z. Han , Y. Lu , Z. Xu , and W. Zhu , “Pentavalent pnictogen bonds involving triarylpnictogen catecholates as strong lewis acids: Crystallographic survey and theoretical analysis,” Computational and Theoretical Chemistry 1248, no. 115 (2025): 171.

[58] J. Sharma , S. R. Dash , and K. Vanka , “Pushing the boundaries of pnictogen‐bonding organocatalysis: A clash of sb(iii) versus bi(iii),” ChemPhysChem 26, no. 16 (2025): e202500265. 40632905 10.1002/cphc.202500265

[59] F. Jiménez‐Grávalos , M. Gallegos , A. Martín Pendás , and A. S. Novikov , “Challenging the electrostatic σ‐hole picture of halogen bonding using minimal models and the interacting quantum atoms approach,” Journal of Computational Chemistry 42, no. 10 (2021): 676–687. 33566376 10.1002/jcc.26488

[60] M. Michalczyk , “Does the presence of sigma holes affect the way neutral ligands attach to a halonium cation?,” ChemPhysChem 26, no. 20 (2025): e2500357. 10.1002/cphc.202500357 40889756

[61] D. Sethio , G. Raggi , R. Lindh , and M. Erdélyi , “Halogen bond of halonium ions: Benchmarking dft methods for the description of nmr chemical shifts,” Journal of Chemical Theory and Computation 16, no. 12 (2020): 7690–7701. 33136388 10.1021/acs.jctc.0c00860 PMC7726912

