# Supplementary material for: Energy and Real Space Characteristics of Non‐Covalent Interactions Across the Periodic Table
Source: J Comput Chem. 2025 Nov 24;46(31):e70268. doi: 10.1002/jcc.70268 (PMC12642420; doi:10.1002/jcc.70268)
Supplement: Supplementary file 1 — Data S1: Supplementary Information. [file JCC-46-0-s001.pdf]

Supporting Information: Energy and real space characteristics of non-covalent interactions across the periodic table

Eline Desmedt [a], Katarzyna Zator [b,*], Tatiana Woller [a], Roberto A. Boto [c], Mercedes Alonso [a], Julia Contreras-García[b,d] *

E-mail: katarzyna.zator@sorbonne-universite.fr,julia.contreras_garcia@sorbonne-universite.fr

[a] *Vrije Universiteit Brussel, ALGC, Pleinlaan 2, Brussels B-1050,Belgium* [b] *Laboratoire de Chimie Théorique, Sorbonne Université and CNRS, 4 Pl Jussieu, F-75005, Paris, France* [c] *Donostia International Physics Center, University of the Basque Country, Paseo de Manuel Lardizábal 4, 20018, Donostia-San Sebastián, Spain* [d] *CNRS*

Geometry of Studied Complexes

HB1

```
4
molecule#1
C          2.27347        0.00088        0.00858
H          3.34732        0.00350        0.02116
C          1.04191       -0.00203       -0.00709
```

```
H          -0.03748       -0.00477       -0.01972
3
molecule#2
O          -2.20780       -0.00068       -0.03849
H          -2.76029        0.76887        0.14711
H          -2.77944       -0.75524        0.15044
```

HB2

```
4
molecule#1
C           0.00000        0.00000        1.68546
H           0.00000        0.00000        2.75895
C           0.00000        0.00000        0.45234
H           0.00000        0.00000       -0.63487
2
molecule#2
Li          0.00000        0.00000       -4.13463
H           0.00000        0.00000       -2.54701
```

HB3

```
2
molecule#1
F           0.01397       -1.06695       -0.00000
H          -0.05296       -0.10340       -0.00000
2
molecule#2
Li          0.01397        2.81823        0.00000
H          -0.11465        1.25128        0.00000
```

HB4

4

molecule#1

C	-1.36326	-0.61528	0.00677
H	-1.36912	-1.69046	0.00100
C	-1.36106	0.61613	0.00689
H	-1.36264	1.69133	0.00134

3

molecule#2

O	1.97618	-0.00035	-0.10610
H	2.25898	-0.00176	0.81671
H	1.00926	-0.00139	-0.05226

HB5

4

molecule#1

C	2.13857	-0.61486	0.00018
H	2.14508	-1.68979	-0.00002
C	2.13792	0.61617	0.00004
H	2.13954	1.69111	0.00027

4

molecule#2

C	-2.75464	0.00103	0.00029
C	-1.52345	-0.00239	-0.00051
H	-0.44644	-0.00486	-0.00119
H	-3.82858	0.00387	0.00097

HB6

```
4
molecule#1
C          0.61585       -1.23587         0.00000
H          1.69150       -1.24281         0.00000
C         -0.61577       -1.23699         0.00000
H         -1.69142       -1.24451         0.00000
2
molecule#2
F         -0.00006        1.82577         0.00000
H          0.00000        0.89258         0.00000
```

P1

```
4
molecule#1
P          1.15054       -0.00354        -0.11002
H          2.51684        0.01472         0.28631
H          0.77534        1.05238         0.75287
H          0.78685       -1.02280         0.79972
4
molecule#2
N         -2.03630       -0.00299         0.03276
H         -2.11034       -0.73169        -0.67119
H         -2.17141        0.88777        -0.43668
H         -2.80128       -0.12634         0.68987
```

P2

```
4
molecule#1
```

P	-1.51348	-0.00000	-0.11803
H	-0.09619	-0.00001	0.02745
H	-1.71942	-1.03998	0.83458
H	-1.71939	1.04000	0.83455

4

molecule#2

N	2.50636	0.00000	0.00726
H	2.89832	-0.00023	-0.93391
H	2.89721	-0.81507	0.47866
H	2.89720	0.81530	0.47826

P3

4

molecule#1

P	1.79188	-0.00022	-0.09440
H	3.21666	0.00052	-0.15384
H	1.73018	1.04168	0.87427
H	1.73135	-1.03897	0.87768

4

molecule#2

P	-1.79189	-0.00021	0.09442
H	-1.73118	-1.03898	-0.87765
H	-1.73004	1.04166	-0.87426
H	-3.21669	0.00049	0.15361

P4

4

molecule#1

P	1.94901	0.00002	-0.12109
H	3.28256	-0.00000	0.38301
H	1.51486	1.03922	0.75121
H	1.51492	-1.03942	0.75096

4

molecule#2

P	-2.10056	-0.00003	-0.02837
H	-1.46146	1.04009	0.70738
H	-1.46119	-1.03916	0.70857
H	-1.11650	-0.00045	-1.05911

P5

4

molecule#1

N	0.75370	-0.92825	0.91887
H	1.57275	-0.87992	0.31139
H	0.04368	-0.31657	0.49747
H	1.03538	-0.49535	1.79901

13

molecule#2

N	-1.91221	0.22873	-0.18589
C	-2.02026	-0.89970	-1.11540
C	-2.44675	1.44429	-0.80012
C	-2.63719	-0.07423	1.04703
H	-1.87673	1.67566	-1.71361
H	-2.34256	2.28855	-0.10145
H	-3.52225	1.34044	-1.06903
H	-2.21931	-0.98263	1.50795

H	-3.72415	-0.23643	0.86843
H	-2.52532	0.76114	1.75565
H	-1.48549	-0.66095	-2.04792
H	-3.07986	-1.13293	-1.36165
H	-1.55846	-1.78799	-0.65888

P6

4

molecule#1

P	0.71416	-0.77814	0.95083
H	0.92081	-0.23968	-0.35251
H	0.41218	0.48130	1.54382
H	2.10473	-0.71888	1.30010

13

molecule#2

N	-1.99327	0.01841	-0.11838
C	-2.19811	-1.05164	-1.09118
C	-2.18023	1.32281	-0.74799
C	-2.90548	-0.14126	1.01160
H	-1.46508	1.43744	-1.57777
H	-1.99229	2.12040	-0.01240
H	-3.20965	1.45394	-1.15298
H	-2.72720	-1.11481	1.49341
H	-3.97351	-0.08949	0.70009
H	-2.72137	0.65558	1.74923
H	-1.48390	-0.93849	-1.92220
H	-3.22887	-1.04865	-1.51257
H	-2.01795	-2.02498	-0.60925

P7

4

molecule#1

As	0.67586	-0.74238	0.94300
H	0.91585	-0.14639	-0.43032
H	0.31502	0.59052	1.56945
H	2.14969	-0.60817	1.33669

13

molecule#2

N	-1.99612	-0.03844	-0.12502
C	-2.26371	-1.08034	-1.11266
C	-2.10275	1.28669	-0.72873
C	-2.90079	-0.16391	1.01413
H	-1.38476	1.37124	-1.55945
H	-1.85833	2.05412	0.02240
H	-3.12306	1.49329	-1.12371
H	-2.76839	-1.15336	1.47896
H	-3.96907	-0.04919	0.72313
H	-2.65911	0.60768	1.76210
H	-1.54146	-0.99685	-1.94005
H	-3.29148	-1.01160	-1.53453
H	-2.14243	-2.06907	-0.64354

P8

4

molecule#1

N	1.44650	-1.13126	0.53244

H	1.95621	-0.85584	-0.30797
H	0.71476	-0.42843	0.66277
H	2.09673	-1.00763	1.30899

13

molecule#2

P	-1.85326	0.42253	0.29883
C	-1.78601	-0.99597	-0.90176
C	-2.57885	1.71914	-0.82682
C	-3.39727	-0.05120	1.22358
H	-1.83390	2.00811	-1.58537
H	-2.83657	2.61806	-0.24444
H	-3.48561	1.35048	-1.33603
H	-3.19915	-0.94567	1.83609
H	-4.22776	-0.26541	0.52951
H	-3.69423	0.76384	1.90129
H	-1.02344	-0.79238	-1.67116
H	-2.75941	-1.15307	-1.39796
H	-1.48378	-1.91143	-0.37014

P9

4

molecule#1

P	1.37454	-1.10355	0.74354
H	1.66560	0.03200	-0.06672
H	1.25622	-0.34660	1.94357
H	2.75048	-1.43453	0.95245

13

molecule#2

P	-1.85524	0.35275	0.11036
C	-1.99350	-1.09501	-1.05587
C	-2.62747	1.65896	-0.97588
C	-3.32261	-0.02146	1.19506
H	-1.95972	1.86797	-1.82725
H	-2.75810	2.59506	-0.40968
H	-3.60818	1.32990	-1.36148
H	-3.09868	-0.89870	1.82291
H	-4.22162	-0.23652	0.59130
H	-3.52563	0.83624	1.85531
H	-1.25319	-1.00023	-1.86648
H	-3.00515	-1.16919	-1.49109
H	-1.76280	-2.02322	-0.50818

P10

4

molecule#1

As	1.29734	-1.15471	0.88158
H	1.55092	-0.23234	-0.29664
H	1.37110	-0.02699	1.88936
H	2.77489	-1.49530	1.03629

13

molecule#2

P	-1.86116	0.36509	0.12510
C	-1.95276	-1.08022	-1.04764
C	-2.63752	1.66637	-0.95804
C	-3.34093	-0.03809	1.18198
H	-1.96623	1.89567	-1.80140

H	-2.77790	2.59199	-0.37718
H	-3.61489	1.33898	-1.35290
H	-3.12539	-0.92166	1.80369
H	-4.23641	-0.23983	0.56884
H	-3.55199	0.80797	1.85562
H	-1.20208	-0.95747	-1.84561
H	-2.95270	-1.16630	-1.50710
H	-1.71932	-2.00931	-0.50410

X1

molecule#1

O	2.75579	0.00001	0.50051
C	3.13378	-1.17184	-0.21636
H	2.63777	-1.20963	-1.20364
H	4.22807	-1.20847	-0.36490
H	2.81800	-2.03437	0.38342
C	3.13387	1.17181	-0.21636
H	2.81832	2.03437	0.38349
H	4.22815	1.20827	-0.36506
H	2.63774	1.20972	-1.20358

5

molecule#2

C	-1.91217	0.00001	-0.04525
Cl	-0.19343	0.00000	0.29165
F	-2.25694	-1.08467	-0.76344
F	-2.62276	-0.00976	1.09650
F	-2.26019	1.09445	-0.74684

X2

9

molecule#1

S	-2.85381	0.00000	-0.61395
C	-2.77764	1.37074	0.58067
H	-3.63970	1.33563	1.26262
H	-1.84044	1.33076	1.15566
H	-2.80910	2.31023	0.01157
C	-2.77767	-1.37074	0.58068
H	-2.80912	-2.31022	0.01160
H	-1.84047	-1.33075	1.15568
H	-3.63973	-1.33561	1.26262

5

molecule#2

C	2.23732	-0.00002	0.05750
Cl	0.55787	-0.00000	-0.44997
F	2.50866	1.08962	0.79824
F	2.50863	-1.08953	0.79839
F	3.05644	-0.00009	-1.00776

X3

9

molecule#1

O	-2.79198	-0.00002	-0.40675
C	-3.29198	1.17403	0.23079
H	-4.39462	1.21009	0.17762
H	-2.98196	1.21043	1.29092
H	-2.87186	2.03546	-0.30279
C	-3.29290	-1.17355	0.23103

H	-2.87379	-2.03542	-0.30263
H	-2.98258	-1.21016	1.29106
H	-4.39560	-1.20852	0.17826

5

molecule#2

C	1.99573	0.00005	0.08459
Br	0.10373	-0.00024	-0.18555
F	2.37926	-1.07542	0.80101
F	2.65032	-0.02885	-1.09261
F	2.38602	1.10464	0.75129

X4

5

molecule#1

C	0.00000	0.00000	2.24796
Br	0.00000	0.00000	0.32890
F	0.00000	1.25953	2.73382
F	1.09078	-0.62976	2.73382
F	-1.09078	-0.62976	2.73382

13

molecule#2

N	0.00000	0.00000	-2.50404
C	0.00000	1.38559	-2.97183
H	0.89267	1.90323	-2.59103
H	-0.89267	1.90323	-2.59103
H	0.00000	1.45078	-4.08159
C	-1.19996	-0.69280	-2.97183
H	-2.09458	-0.17854	-2.59103

```
H          -1.20191        -1.72469        -2.59103
H          -1.25641        -0.72539        -4.08159
C           1.19996        -0.69280        -2.97183
H           1.20191        -1.72469        -2.59103
H           1.25641        -0.72539        -4.08159
H           2.09458        -0.17854        -2.59103
```

X5

```
5
molecule#1
C           0.00000         0.00000         2.77465
F          -1.09042        -0.62955         3.25647
F           1.09042        -0.62955         3.25647
F           0.00000         1.25910         3.25647
Br          0.00000         0.00000         0.85873
13
molecule#2
P           0.00000         0.00000        -2.59938
C           0.00000         1.63316        -3.48501
H          -0.89027         2.21151        -3.19482
H           0.89027         2.21151        -3.19482
H           0.00000         1.49703        -4.57938
C           1.41435        -0.81658        -3.48501
H           2.36036        -0.33476        -3.19482
H           1.47009        -1.87675        -3.19482
H           1.29646        -0.74851        -4.57938
C          -1.41435        -0.81658        -3.48501
H          -1.47009        -1.87675        -3.19482
```

H	-1.29646	-0.74851	-4.57938
H	-2.36036	-0.33476	-3.19482

X6

5

molecule#1

C	0.00000	0.00000	2.36592
F	0.00000	1.26122	2.86259
F	1.09225	-0.63061	2.86259
F	-1.09225	-0.63061	2.86259
I	0.00000	0.00000	0.20659

13

molecule#2

N	0.00000	0.00000	-2.61141
C	0.00000	1.38861	-3.07853
H	0.89286	1.90463	-2.69669
H	-0.89286	1.90463	-2.69669
H	0.00000	1.44906	-4.18715
C	-1.20257	-0.69431	-3.07853
H	-2.09589	-0.17908	-2.69669
H	-1.20303	-1.72556	-2.69669
H	-1.25492	-0.72453	-4.18715
C	1.20257	-0.69431	-3.07853
H	1.20303	-1.72556	-2.69669
H	1.25492	-0.72453	-4.18715
H	2.09589	-0.17908	-2.69669

X7

```
9
molecule#1
C          4.54427       0.68924      -0.00032
C          4.54478      -0.68819      -0.00054
H          5.36503       1.39929      -0.00048
H          5.36607      -1.39763      -0.00092
N          3.20791      -1.05755      -0.00014
H          2.87697      -2.01457      -0.00017
N          3.20713       1.05760       0.00016
H          2.87550       2.01439       0.00040
C          2.33634      -0.00029       0.00032
5
molecule#2
C         -2.68850       0.00017      -0.00021
F         -3.19594       0.00070       1.26103
F         -3.19317       1.09339      -0.63174
F         -3.19538      -1.09223      -0.63110
I         -0.51981      -0.00046       0.00041
```
